# Construction of a HSC activation-related lncRNA–miRNA–mRNA ceRNA regulatory network reveals potential molecules involved in liver fibrosis

**DOI:** 10.3389/fgene.2025.1640326

**Published:** 2025-11-10

**Authors:** Kaiqiang Wan, Qiong Zhou, Ruirui Feng, Tao Liu, Chang Fan, Xue Pang, Zhongliang Li, Qiumei Zhou

**Affiliations:** 1 Experimental Center of Clinical Research, The First Affiliated Hospital of Anhui University of Chinese Medicine, Hefei, China; 2 School of Pharmacy, Anhui University of Chinese Medicine, Hefei, China; 3 Department of Laboratory Medicine, The First Affiliated Hospital of Anhui University of Chinese Medicine, Hefei, China

**Keywords:** competitive endogenous RNA, liver fibrosis, hepatic stellate cells, whole transcriptome sequencing, long noncoding RNA

## Abstract

**Background:**

Liver fibrosis (LF) represents a progressive pathophysiological consequence of persistent liver injury. Although the competitive endogenous RNA (ceRNA) network serves as a critical regulator in diverse disease pathogenesis, its molecular underpinnings in LF and fibrogenic mediators remain unknown.

**Objective:**

In this study, we aimed to systematically probe the LF-related ceRNA regulatory axis and identify the potential molecules involved in the activation of hepatic stellate cells (HSCs).

**Methods and Results:**

Based on the whole transcriptome RNA sequencing, 401 lncRNAs, 60 miRNAs, and 1,224 mRNAs were identified between model and normal liver tissue samples. Then, through target gene prediction, an lncRNA–miRNA–mRNA (LMM) ceRNA network comprising four differentially expressed lncRNAs (DE lncRNAs), six DE miRNAs, and 148 DE mRNAs was established. The expression levels of these RNAs were verified by RT-qPCR. Functional annotation via the Kyoto Encyclopedia of Genes and Genomes (KEGG) pathway enrichment analysis revealed that target mRNAs of co-dysregulated lncRNAs and miRNAs in model groups were significantly enriched in multiple pathways, such as unsaturated fatty acids and TGF-β signaling pathways. Notably, four hub mRNAs (HMGCR, SREBF-1, TGF-β3, and FBN1) were identified by constructing a protein–protein interaction (PPI) network with the 148 DE mRNAs. Importantly, the dual-luciferase reporter assay confirmed the existence of specific binding sites among lncRNA H19, miR-148a-3p, and FBN1. Finally, the gene expression levels were verified by RT-qPCR in TGF-β1-induced JS-1 cells, revealing that five lncRNA–miRNA–mRNA relationship pairs containing H19, miR-130a-3p, miR-148a-3p, TGF-β3, FBN1, and HMGCR were involved in the activation of HSCs.

**Conclusion:**

In this study, an HSC activation-related ceRNA network was successfully established in mice liver tissue, which could provide a novel framework for elucidating pathogenic mechanisms and identifying clinically relevant prognostic markers in LF progression.

## Graphical Abstract

**Figure:**
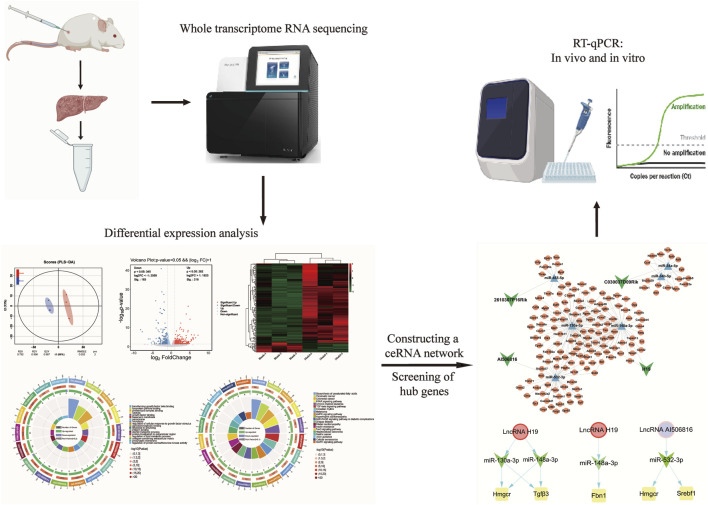

## 1 Introduction

Liver fibrosis (LF) is caused by chronic liver injury, resulting in an abnormal wound-healing response, stemming from issues such as alcoholism, viral infections, autoimmune diseases, and other liver diseases, which can develop into cirrhosis or even liver cancer (Zhao et al., 2023; Zhang et al., 2023). It seriously affects human health and has become a highly morbid medical problem. LF manifests as a pathological cascade centered on the activation of hepatic stellate cells (HSCs), with concomitant pathological extracellular matrix (ECM) deposition driving progressive architectural reorganization (Dewidar et al., 2019; Lai et al., 2024; Yao et al., 2024). HSCs are a key bridge in the development of LF and undergo phenotypic changes from a quiescent state to activated proliferative myofibroblasts (Zhou et al., 2024a; Zhang et al., 2024a). However, current research has not fully delineated the molecular checkpoints capable of interrupting abnormal activation of HSCs and halting fibrotic cascade progression (Baek et al., 2024). Therefore, the systematic elucidation of pathway-specific molecular determinants controlling the pathophysiological cascade of LF is an imperative focus of translational research, thereby providing significant information for the development of pharmacological interventions in the treatment of LF.

The competing endogenous RNA (ceRNA) interactome constitutes an intricate regulatory framework that includes both coding and noncoding RNA species (lncRNAs, circRNAs, miRNAs, mRNAs, and pseudogenes) and represents one of the most critical phenomena in the molecular mechanism for regulating homeostatic post-transcriptional modulation in cells (Wang et al., 2016; Khashkhashi Moghadam et al., 2022; Yan and Bu, 2021). Although the emerging evidence underscores the key regulatory effects of the lncRNA–miRNA–mRNA (LMM) axis in orchestrating hepatic fibrogenesis initiation and progression, the full extent and mechanisms of its involvement remains under investigation (Sun et al., 2024a; Cui et al., 2024). Most lncRNAs can act as sponges of miRNA to regulate gene expression, which is a key to elucidating the molecular mechanism of lncRNAs (Kumar et al., 2024). Previous studies show that lncRNA Neat1 can promote LF progression by acting on its downstream target miR-148a-3p and miR-22-3p and further regulating the lncRNA Neat1/miR-148a-3p and miR-22-3p/Cyth3 network pathways (Huang et al., 2021). Interestingly, one study reported that miR-148a-3p regulates alcoholic LF by targeting ERBB3, suggesting that miR-148a-3p is closely associated with the progression of LF (Xiong et al., 2020). Another study also showed that the deletion of lncRNA Gpr137b-ps inhibited HSC activation and ameliorated LF in mice by regulating miR-200a-3p, providing a new therapeutic target for patients with LF (Liao et al., 2021). However, uncharacterized ceRNA-regulated lncRNAs may regulate the pathogenesis and progression of LF, which warrants systematic investigation of their mechanistic contributions.

In this study, we performed a comparative transcriptome assay of liver tissue using whole transcriptome sequencing to characterize the differential expression patterns of lncRNAs, miRNAs, and mRNAs between mouse models of LF and controls and identified the molecular regulatory mechanisms of the LMM ceRNA network for the process of LF. Transcriptome investigation indicated 401 lncRNAs, 60 miRNAs, and 1,224 mRNAs with differential expression profiles. Through computational integration, a nuclear ceRNA network comprising four lncRNAs, six miRNAs, and 148 mRNAs was established, which constitute the functional backbone of fibrotic signaling. Thereafter, functional enrichment analyses (Gene Ontology and KEGG) were conducted to elucidate molecular pathways underlying hepatic fibrogenesis pathogenesis. In order to identify key biomarkers, we constructed the protein–protein interaction (PPI) network to screen for core genes out of 148 mRNAs. Finally, biomarkers closely associated with HSC activation were validated by RT-qPCR in transforming growth factor beta (TGF-β1)-induced JS-1 cells.

## 2 Materials and methods

### 2.1 Mice model

Male C57BL/6 mice (SPF, 20 ± 2 g, 6–8 weeks) were purchased from Hangzhou Ziyuan Laboratory Animal Co. (SCXK (Zhe) 2019-0004) and housed under controlled conditions (22 °C ± 2 °C, 60% ± 5% humidity, and 12 h light/dark cycle) at the First Affiliated Hospital of Anhui University of Chinese Medicine, China. All animal experiments performed in this study were approved by the Experimental Animal Ethics Committee of Anhui University of Chinese Medicine (approval no. AHUCM-mouse-2021046) and conducted in compliance with the guidelines established by China’s National Health and Medical Research Council. Animal studies are reported in accordance with ARRIVE guidelines. After 1 week of acclimatization, the mice were divided into control (n = 3) and model (n = 3) groups. According to our previous study (Pang et al., 2024), LF was induced in mice in the model group, which were dorsally injected subcutaneously with a mixture of CCl_4_ and olive oil (v/v = 1:4), 0.1 mL/10 g twice a week for 12 weeks. Meanwhile, the control group was given an equal amount of olive oil as a contrast. At the end of modeling, they were anesthetized by abdominal injection of sodium pentobarbital. Subsequently, the mice were subjected to cervical dislocation, and dissection was performed after confirming that their pupils were dilated and their heartbeat had stopped. In brief, the mice were fixed on a dissection board, sterilized with 75% alcohol all over the body, and the skin and peritoneum were cut along the median line of the abdomen to expose the liver. Adequate anesthesia was induced during liver tissue extraction using the above drugs to avoid suffering of the animals. The collected liver tissue samples from each group were cut into partial tissue blocks, encapsulated in cryotubes, labeled with the group, and stored at −80 °C for subsequent analysis.

### 2.2 Histopathological assessment

The removed mouse liver tissues were fixed in 4% FPA. Subsequently, paraffin-embedded sections were cut into homogeneous 4 μm sections, which were subjected to hematoxylin and eosin (HE) staining and Masson staining. Then, the histological assessment was conducted by HE staining, and the collagen deposition evaluation was conducted by Masson staining.

### 2.3 Immunohistochemical detection

The liver tissue sections underwent heat-induced antigen retrieval by pressurization (in pH 7.0 buffer at 121 °C for 3 min) to expose epitopes. Endogenous peroxidase activity was blocked with 3% H_2_O_2_ (20 min, RT). After rinsing thrice with PBS (5 min/repeat), nonspecific binding sites were depressed with 10% normal goat serum (20 min, RT). Then, the primary antibodies, namely, α-SMA (1:300, AF1032, Affinity) and Collagen I (1:400, AF7001, Affinity), were incubated in a humidified chamber (37 °C, 60 min), followed by three PBS-T (0.05% Tween 20) washes (3 × 5 min). Subsequently, the secondary antibody (1:300) was added (37 °C, 20 min). Finally, chromogenic detection utilized DAB substrate with reaction termination under bright-field microscopy.

### 2.4 Transcriptome sequencing analysis

The total RNA profiles were extracted from mouse liver tissue (control vs. fibrosis model, n = 3/group) using TRIzol^®^ reagent (Thermo Fisher Scientific, United States), and RNA integrity was quantitatively confirmed by an Agilent 2100 Bioanalyzer (Agilent Technologies, China); for mRNA, lncRNA, and miRNA sequencing, a total of six cDNA libraries (Blank-1, Blank-2, Blank-3, Model-1, Model-2, and Model-3) were constructed from the control and model groups. As described by Zhang et al. (2024b), cDNA library construction and Illumina HiSeq 2000 sequencing were conducted at BGI (Shenzhen, China).

### 2.5 Differential expression analysis

Transcript quantification was normalized using FPKM, and DE lncRNAs, DE miRNAs, and DE mRNAs were identified by significance thresholds |log2FoldChange| >1 and p-value <0.05 in model groups compared to the control groups. Then, volcano and heatmap were used to identify the intersection of DE RNAs, based on the Ouyi Group Cloud Platform. Functional enrichment analysis of intersecting DE RNAs was performed using GO and KEGG databases, with a significance threshold of p < 0.05 for pathway annotation.

### 2.6 Construction of the lncRNA–miRNA–mRNA ceRNA network

The ceRNA regulatory axis originates from the molecular sponge paradigm, where lncRNAs sequester miRNAs to post-transcriptionally modulate target mRNA expression (Fan et al., 2020). First, the regulatory relationship was searched from the STARBASE (https://rnasysu.com/encori/index.php) as DE lncRNA–DE miRNA pairs and DE miRNA–DE mRNA pairs. Among the predicted pairs, both DE lncRNA and DE mRNA are concurrently targeted by the same DE miRNA and display a negative co-expression. Finally, the lncRNA–miRNA–mRNA ceRNA network was built and visualized using Cytoscape 3.8.2 software. Based on (https://www.omic share.com) online software, GO analysis and KEGG pathway enrichment were conducted to functionally annotate, visualize, and integratively identify biological processes and signaling pathways associated with the ceRNA network’s DE mRNAs at a significant threshold of p < 0.05.

### 2.7 Construction of the protein–protein interaction network

A PPI network for DE mRNAs in the ceRNA regulatory axis was established through STRING database analysis (https://cn.string-db.org/). A confidence value > 0.4 was considered to be significant. Hub genes within the PPI interactome were prioritized through topological centrality metrics (maximal clique centrality, maximum neighborhood component, and degree algorithms, respectively) and visualized using the CytoHubba plugin in Cytoscape 3.8.2. The standard for the definition of the core genes of DE mRNA in the ceRNA network is that genes should be simultaneously recognized by all three algorithms.

### 2.8 Validation of RT-qPCR

Total RNA isolation from mouse liver tissues was performed utilizing TRIzol reagent methodology, with RNA integrity verified via UV-vis spectrophotometry (A260/A280 ratio >1.8). Reverse transcription of lncRNAs, miRNAs, and mRNAs was performed using manufacturer-specified reverse transcription protocols, and the primer sequences for all analyzed transcripts are detailed in Table 1. In order to confirm the specificity of the PCR products, we plotted the relevant melt curves and determined the number of cycles required and the Ct values. All lncRNAs, miRNAs, and mRNAs were replicated for validation, and results were expressed using 2^−ΔΔCt^ values. The gene levels were estimated using the t-test, and a p-value <0.05 was considered significant.

**TABLE 1:** Sequences of primers used for RT-qPCR.

Gene	Category	Primer sequence (5′–3′)
AI506816	lncRNA	F:TGCAATGAGAATGCCTCAAAAT R:GTCTCTCCAGCCCTAGAGTCAAG
H19	lncRNA	F:GCAGAGAAGTGTTAGCTCTTTGGG R:TTCTTGAACACCATGGGCTGG
C030037D09Rik	lncRNA	F:CAGTCATTTTGAACTGGAGGTGAG R:CTGGTGTTTTGGCTAACGAGTTG
2610307P16Rik	lncRNA	F:CGAGTTTCGTCTTCCTGTTTTGAC R:GACATTCGTGACTCCCAGATGTAC
miR-34a-5p	miRNA	F:AGTGCAGGGTCCGAGGTATT R:CGCGTGGCAGTGTCTTAGCT
miR-34b-5p	miRNA	F:CGCGAGGCAGTGTAATTAGCT R:AGTGCAGGGTCCGAGGTATT
miR-130a-3p	miRNA	F:AGTGCAGGGTCCGAGGTATT R:CGCGCAGTGCAATGTTAAAA
miR-148a-3p	miRNA	F:AGTGCAGGGTCCGAGGTATT R:GCGCGTCAGTGCACTACAGAA
miR-455-5p	miRNA	F:CGCGTATGTGCCTTTGGACT R:AGTGCAGGGTCCGAGGTATT
miRNA-532-3p	miRNA	F:GCCTCCCACACCCAAGG R:AGTGCAGGGTCCGAGGTATT
U6	miRNA	F:GCTCGCTTCGGCAGCACATATAC R:AGTGCAGGGTCCGAGGTATT
α-SMA	mRNA	F:GTCCCAGACATCAGGGAGTAA R:TCGGATACTTCAGCGTCAGGA
Collagen Ⅰ	mRNA	F:AGCACTCGCCCTCCCGTCTT R:CAATGGCACGGCTGTGTGCG
EGFR	mRNA	F:GGACTGTGTCTCCTGCCAGAAT R:GGCAGACATTCTGGATGGCACT
Ncam1	mRNA	F:GGTTCCGAGATGGTCAGTTGCT R:CAAGGACTCCTGTCCAATACGG
Scn8a	mRNA	F:CACAGAGGATGTTAGCAGCGAG R:CTTCCACCTCAGGCTTGATGTC
Map1b	mRNA	F:AAGTCTGCTCTTCGTGATGCTTAC R:GGATGGACTCTTGGCTGGG
Nedd4l	mRNA	F:AAGTCATAAATCTCGAGTCAAGGG R:TCTTCATCCTGACCTCCGTTTT
Serpine1	mRNA	F:CCTCTTCCACAAGTCTGATGGC R:GCAGTTCCACAACGTCATACTCG
Cd36	mRNA	F:GGACATTGAGATTCTTTTCCTCTG R:GCAAAGGCATTGGCTGGAAGAAC
Kcnma1	mRNA	F:CCTGAAGGACTTTCTGCACAAGG R:ACTCCACCTGAGTGAAATGCCG
Fbn1	mRNA	F:TGTATTGTTCCCATTTGCCGG R:GGAAGGAGATATCTGACCAGACGG
HMGCR	mRNA	F:ACAAGCAGAGACAGAATCGACAC R:TCTGGTTCCTTCTCACAAGCAG
TGF-β3	mRNA	F:AAGCAGCGCTACATAGGTGGCA R:GGCTGAAAGGTGTGACATGGAC
Srebf1	mRNA	F:CGACTACATCCGCTTCTTGCAG R:CCTCCATAGACACATCTGTGCC
β-actin	mRNA	F:AGTGTGACGTTGACATCCGT R:TGCTAGGAGCCAGAGCAGTA

### 2.9 Cell culture

JS-1 cells (Hunan FengHui Biotechnology, CL0417) were maintained in Dulbecco’s Modified Eagle Medium (DMEM) supplemented with 10% heat-inactivated fetal bovine serum (FBS) and 1% antibiotic–antimycotic cocktail (100 U/mL penicillin G, 100 μg/mL streptomycin sulfate) under defined atmospheric parameters (37 °C, 5% CO_2_, 90% relative humidity). In order to induce a cellular model of LF, serum-starved cells were treated with 1 ng/mL recombinant TGF-β1 for 24 h following 24-h serum deprivation.

### 2.10 CCK-8 assay

JS-1 cells were plated at a density of 5 × 10^3^ cells/well in 96-well microplates and maintained in complete culture medium for 24 h stabilization. Following serum starvation (0% FBS, 24 h), cells were exposed to recombinant human TGF-β1 at escalating concentrations (0.1 ng/mL–10 ng/mL) in six replicate wells for 24 h under 5% CO_2_ at 37 °C. Cell viability was quantified using the Cell Counting Kit-8 (CCK-8), using 10 μL/well reagent incubated for 60 min prior to spectrophotometric detection at λ = 450 nm.

### 2.11 Luciferase assays

Using bioinformatic tools (https://rnasysu.com/encori/), we predicted the binding sites between lncRNA H19 and its putative target miR-148a-3p and between miR-148a-3p and its target gene FBN1. Based on these predictions, overexpression plasmids were constructed, and a luciferase reporter assay was employed to validate the binding activity of miR-148a-3p. For the assay, 293T cells were seeded in 96-well plates and allowed to reach 70% confluence, followed by transfection with miR-148a-3p mimic using Lipofectamine 3000 reagent.

### 2.12 Statistical analysis

All statistical computations were conducted using GraphPad Prism 8.0.1, with experimental data presented as the mean ± SD (standard deviation). Intergroup comparisons employed Student’s t-test for dual cohorts and one-way ANOVA for multi-group analyses, applying a significance threshold of p < 0.05. All experiments involved in this study were independently repeated at least thrice to ensure reproducibility.

## 3 Results

### 3.1 Mouse liver fibrosis model was successfully constructed

**FIGURE 1:**
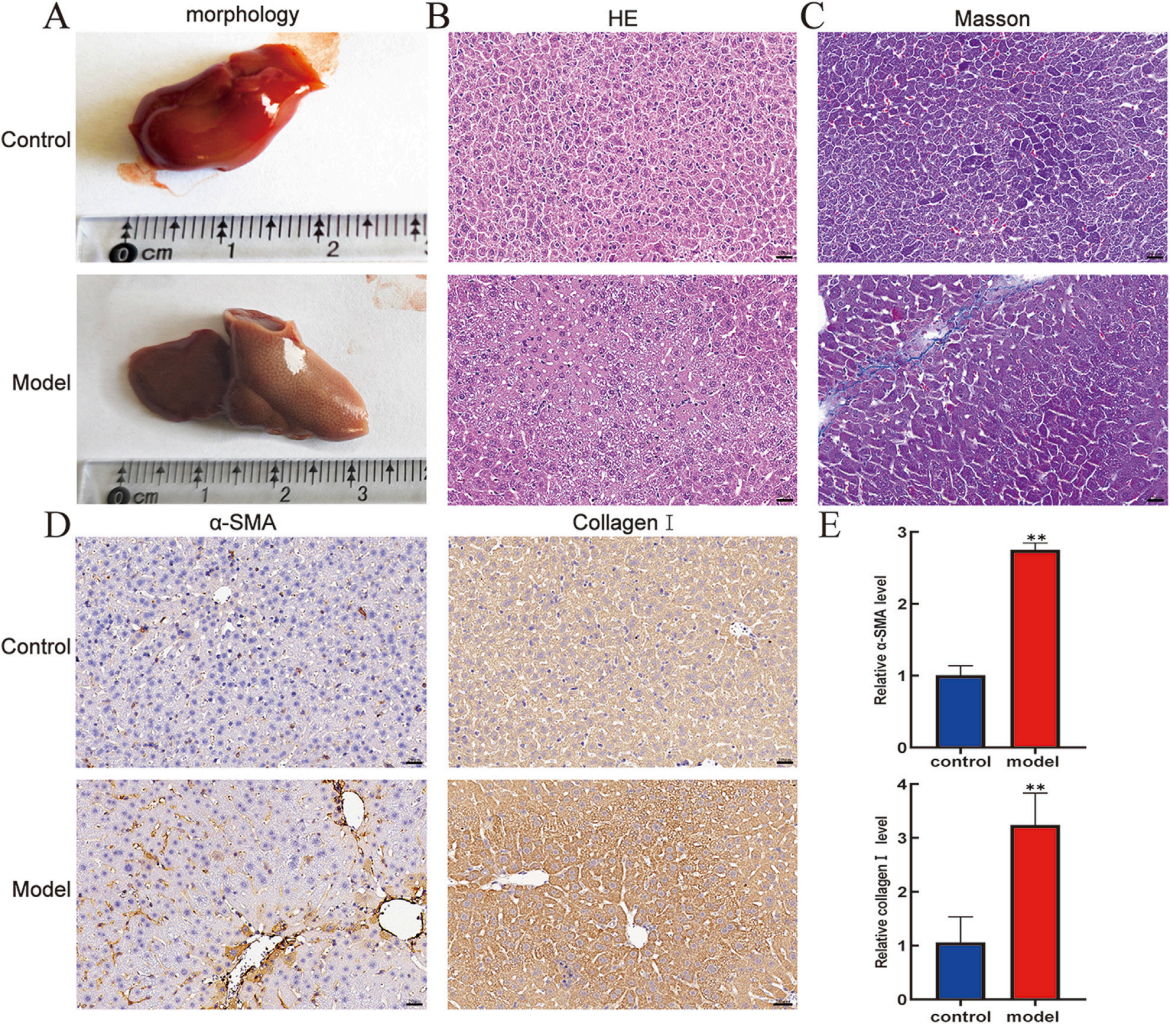
Pathologic livers of mice were analyzed by morphology, HE, Masson, and immunohistochemistry. **(A)** Morphology. **(B)** HE staining (400×). **(C)** Masson staining (400×). Scale bar is 20 μm. **(D)** Protein expression levels of α-SMA and collagen I in mouse livers. **(E)** mRNA expression level of α-SMA and collagen Ⅰ in mouse livers. Compared with the control group, **p < 0.01.

The differences in liver appearance between control groups and model groups were detected by observing liver entities. As shown in Figure 1A, compared with that of the control group, the liver of the model group was larger in size, had a rougher surface, and had more irregular nodules. Furthermore, HE staining was used to define the pathological injury of mouse liver, showing that the liver lobule structure of the model group was damaged, and extensive inflammatory cell infiltration was observed in the portal vein region (Figure 1B). Moreover, Masson staining showed excessive deposition of collagen fibers in the pericentral and periportal vein areas of the liver tissue in the model group compared to that in the control group (Figure 1C). Subsequently, to further verify the activation status of HSCs in mice liver tissue, immunohistochemistry was used to examine the expression levels of α-SMA and collagen I in liver tissue, showing that the expression levels of α-SMA and collagen I were higher in the model group than in the control (Figure 1D). Expectedly, RT-qPCR results confirmed similar expression levels as described in the immunohistochemistry description (Figure 1E). These findings suggest that CCl_4_ successfully structures pivotal pathological features of liver injury and fibrosis progression in a mouse model.

### 3.2 Analysis of differentially expressed lncRNA–miRNA pairs

**FIGURE 2:**
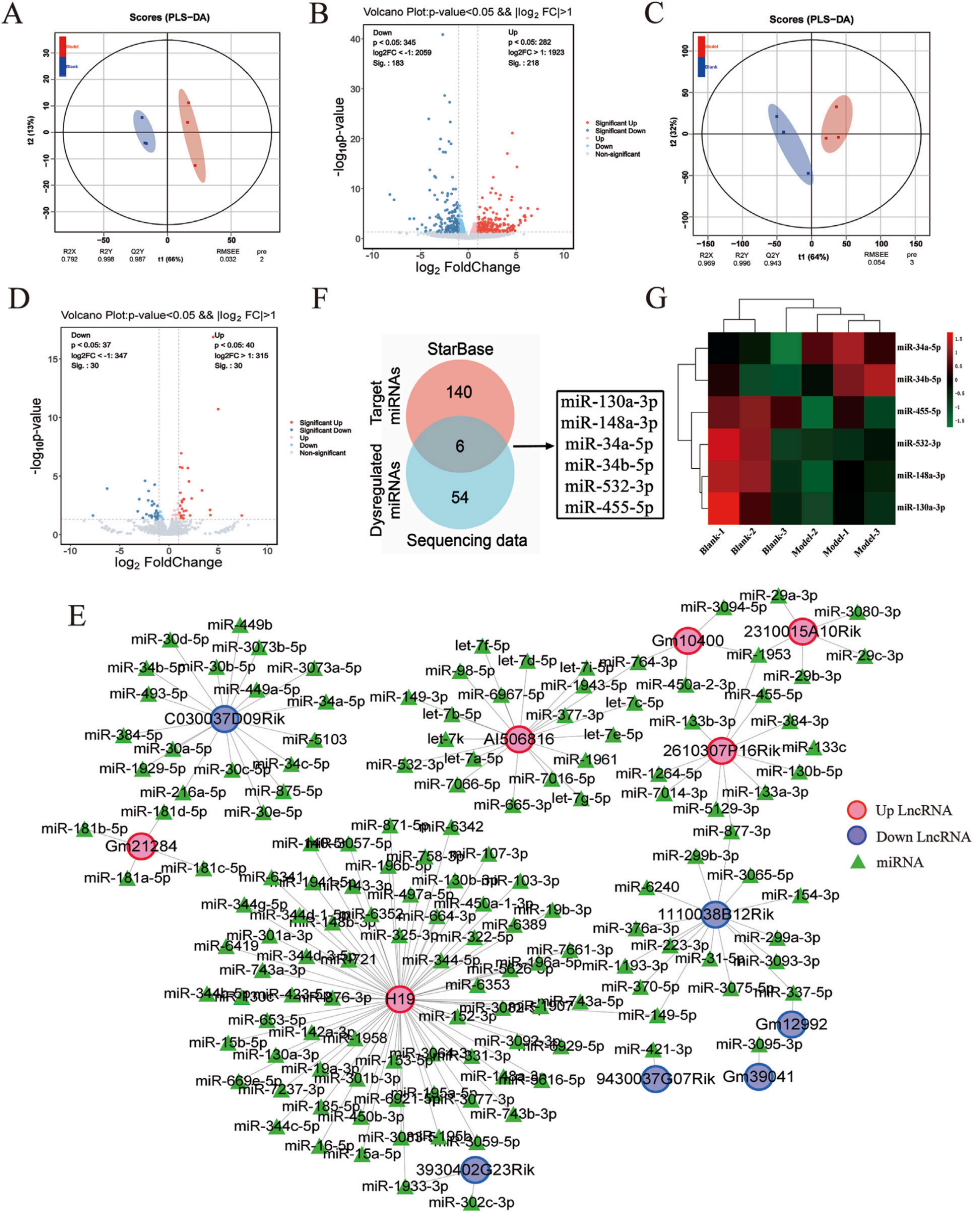
DE LncRNAs–miRNAs pairs analysis. **(A)** Principal component analysis plot of DE lncRNA expression profiles. **(B)** Volcano plot of DE lncRNA expression profiles between control and model groups, where red represents upregulation and blue represents downregulation. **(C)** Principal component analysis plot of DE miRNA expression profiles. **(D)** Volcano plot of DE miRNA expression profiles between control and model groups, with red representing upregulation and blue representing downregulation. **(E)** DE lncRNAs–miRNAs pairs targeting relationship. **(F)** Venn diagram of targeted miRNAs versus DE miRNAs. **(G)** Heat map of the six miRNAs.

To investigate the molecular events underlying the LF process, RNA-seq was performed to examine the transcriptional profiles in the control- and model-group mice. The RNA-seq count data from six samples are shown in Supplementary Table S1, Additional file 1. To assess the consistency of the samples, we performed the principal component analysis (PCA). As shown in Figures 2A, C, lncRNA and miRNA profiles exhibited high within-group clustering and greater intergroup differences than intragroup differences. Transcriptomic profiling revealed 401 DE lncRNAs in fibrotic murine models versus controls (p < 0.05, |log2FoldChange|>1), comprising 54.4% upregulated (n = 218) and 45.6% downregulated (n = 183) transcripts, demonstrating significant noncoding RNA dysregulation during fibrogenesis (Figure 2B). Additionally, 60 differentially expressed miRNAs (DE miRNAs) were identified in the control and model groups, with 30 upregulated and 30 downregulated DE miRNAs (p < 0.05, |log2FC|>1) (Figure 2D). Following bioinformatic processing, volcano plot construction employing the pheatmap package facilitated differential expression visualization, with 401 DE lncRNAs and 60 DE miRNAs meeting quantitative thresholds for subsequent mechanistic interrogation.

Based on the relationships in ceRNA theory, we searched for lncRNA–miRNA interactions using the STARBASE database. As shown in Figure 2E, a total of 146 relevant targets were obtained. Furthermore, we assessed the intersection of DE miRNAs with the targeted miRNAs and identified six overlapping DE miRNAs (Figure 2F). Additionally, six miRNAs were significantly different in the control and model groups as key miRNAs (Figure 2G). These results showed that six lncRNA–miRNA interaction pairs were obtained by STARBASE database prediction, and their targeting relationships are shown in Table 2.

**TABLE 2:** Targeting of DE lncRNAs–DE miRNAs pairs.

lncRNA	Log2(FC)	P-value	Level	miRNA	Log2(FC)	P-value	Level
AI506816	1.31704	0.00057	Up	miR-532-3p	−2.28187	0.01824	Down
C030037D09Rik	−1.16817	0.00049	Down	miR-34a-5p	1.12671	1.73128E-11	Up
				miR-34b-5p	1.46310	0.04133	Up
H19	3.81503	0.00013	Up	miR-130a-3p	−1.71790	0.00316	Down
				miR-148a-3p	−1.12986	0.02028	Down
2610307P16Rik	2.48229	8.91626E-11	Up	miR-455-5p	−1.44659	0.00005	Down

### 3.3 LncRNA–miRNA–mRNA ceRNA network construction

**FIGURE 3:**
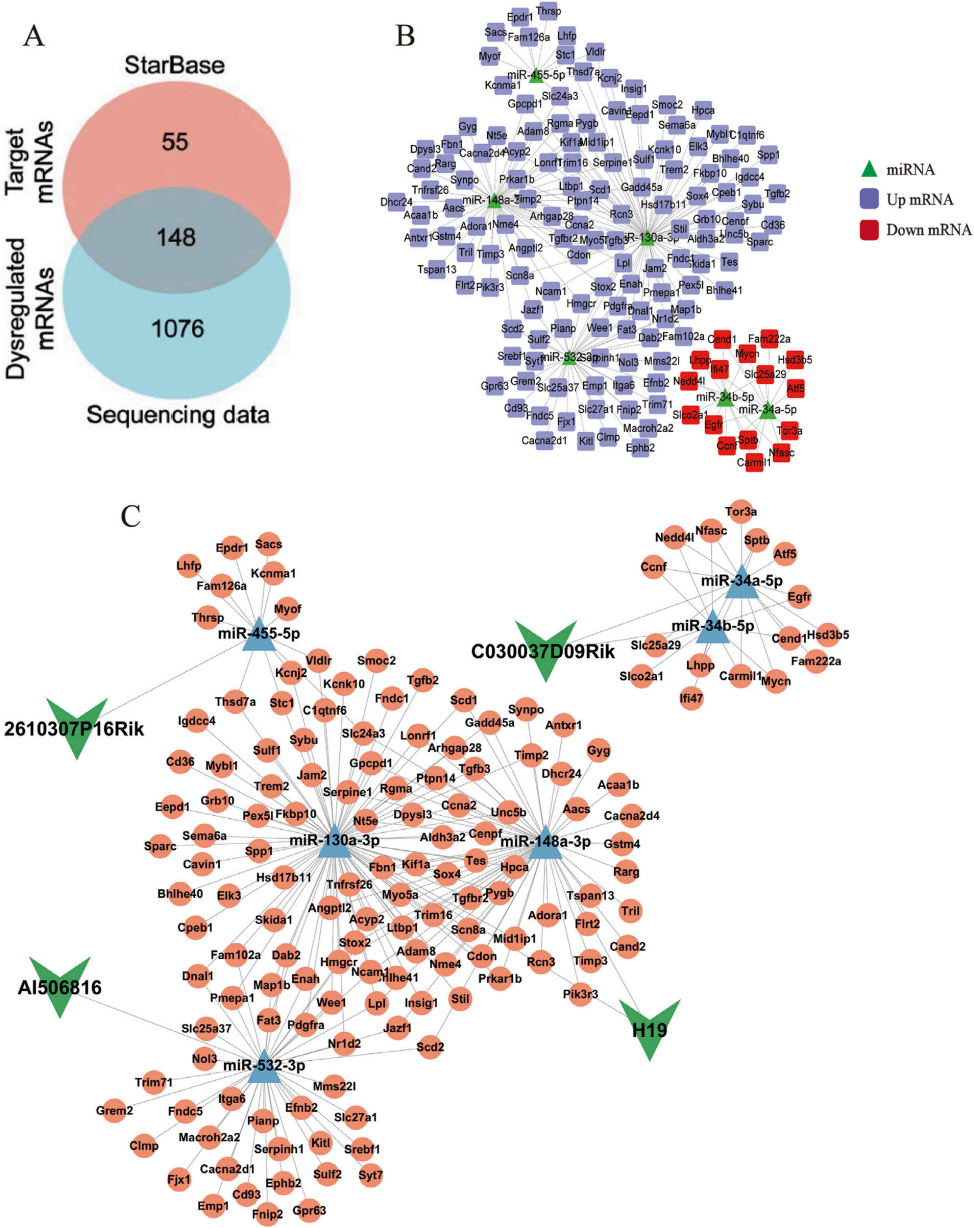
LncRNA–miRNA–mRNA interaction network. **(A)** Venn diagram of targeted mRNAs versus DE mRNAs. **(B)** Network diagram of DE miRNAs–DE mRNAs pairs. The green triangles represent miRNAs, the purple squares represent upregulated mRNAs, and the red squares represent downregulated mRNAs. **(C)** LncRNA–miRNA–mRNA ceRNA network. The green arrows represent lncRNAs, the blue triangles represent miRNAs, and the pink circles represent mRNAs.

LncRNAs act as competitive endogenous RNA sponges through sequence-specific sequestration of miRNA binding sites, effectively modulating the post-transcriptional availability of miRNA-targeted mRNAs within cellular regulatory networks. Therefore, the downstream target mRNA of six DE lncRNA–DE miRNA pairs were searched using the STARBASE database. Furthermore, as described in our previous study, a total of 1,224 differentially expressed mRNAs (DE mRNAs) were identified in the control and model groups (p < 0.05, |log2FoldChange| >1) (Zhang et al., 2024b). After intersection analysis of database-derived mRNAs, 148 DE mRNAs were eventually identified as downstream target genes of the six DE lncRNA–DE miRNA pairs (Figure 3A), and the DE miRNA–DE mRNA regulatory network is identified in Figure 3B. Through systemic integration of lncRNA–miRNA–mRNA interactome data, we depicted a network of ceRNAs including four DE lncRNAs, six DE miRNAs, and 148 DE mRNAs (Figure 3C). This regulatory framework illuminates new mechanistic dimensions of LF.

### 3.4 Functional analysis of the ceRNA network-associated DE mRNAs and PPI network construction

**FIGURE 4:**
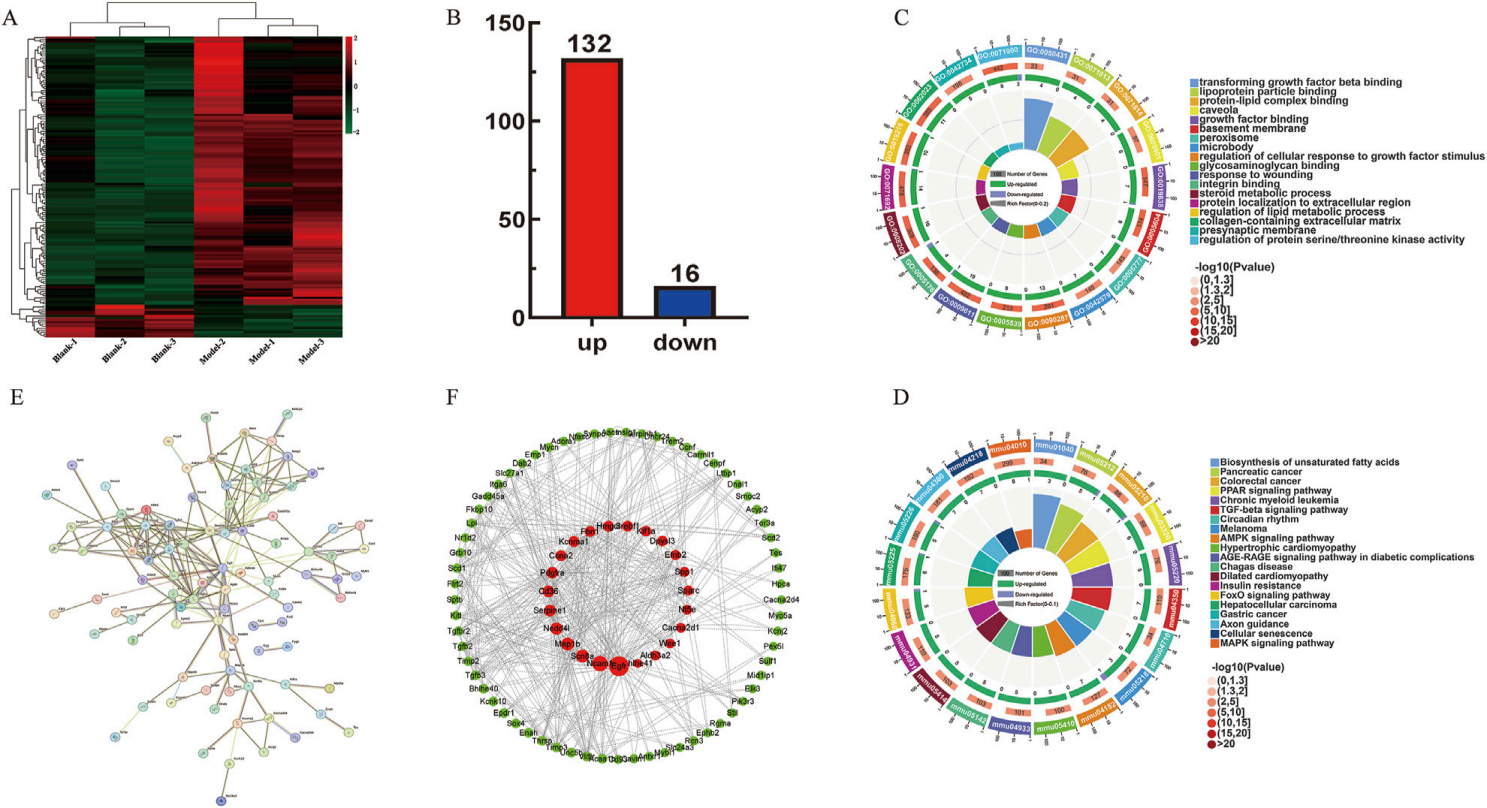
ceRNA network-associated DE mRNAs functional analysis. **(A)** Heatmap of 148 DE mRNAs between control and model groups. **(B)** Up- and downregulation of 148 DE mRNAs, where red represents upregulation and blue represents downregulation. **(C)** GO enrichment analysis plot of 148 DE mRNAs. **(D)** KEGG enrichment analysis of 148 DE mRNAs. **(E,F)** Protein–protein interaction network diagram. The red circle represents the core genes.

The expression of 148 DE mRNAs was visualized by hierarchical clustering analysis (Figure 4A), including 132 upregulated and 16 downregulated mRNAs (Figure 4B). Further analysis of these 148 significant DE mRNAs was conducted by annotating and categorizing. GO analysis revealed that biological processes (BP) were mainly related to the regulation of cellular response to growth factor stimulus, protein localization to extracellular region, response to wounding, and regulation of the lipid metabolic process. The main cellular component (CC) analysis revealed predominant localization to the collagen-containing extracellular matrix, basement membrane structures, and intrinsic presynaptic membrane constituents. Molecular function (MF) is mainly glycosaminoglycan binding, integrin binding, growth factor binding, and transforming growth factor binding. Taken together, Figures 4C, D delineate the 20 most significantly enriched biological processes (BP) and KEGG terms, respectively. KEGG pathway analyses revealed that biosynthesis of unsaturated fatty acids, pancreatic cancer, TGF-β signaling pathway, AMPK signaling pathway, and hepatocellular carcinoma may be essential pathways involved in the pathogenesis of fibrosis. Particularly, the TGF-β signaling pathway was tightly associated with LF, which is shown in Supplementary Figure S1E. Subsequently, interaction relationships for the 148 DE mRNAs were extracted, and PPI network was constructed via the STRING repository. Then, the network topology was analyzed using Cytoscape (v3.8.2), with node diameters scaled by connectivity (Figure 4E). Interactions exceeding a confidence threshold of 0.4 (STRING database) were retained for subsequent analysis (Figure 4F).

### 3.5 Validation of the ceRNA network RNAs

**FIGURE 5:**
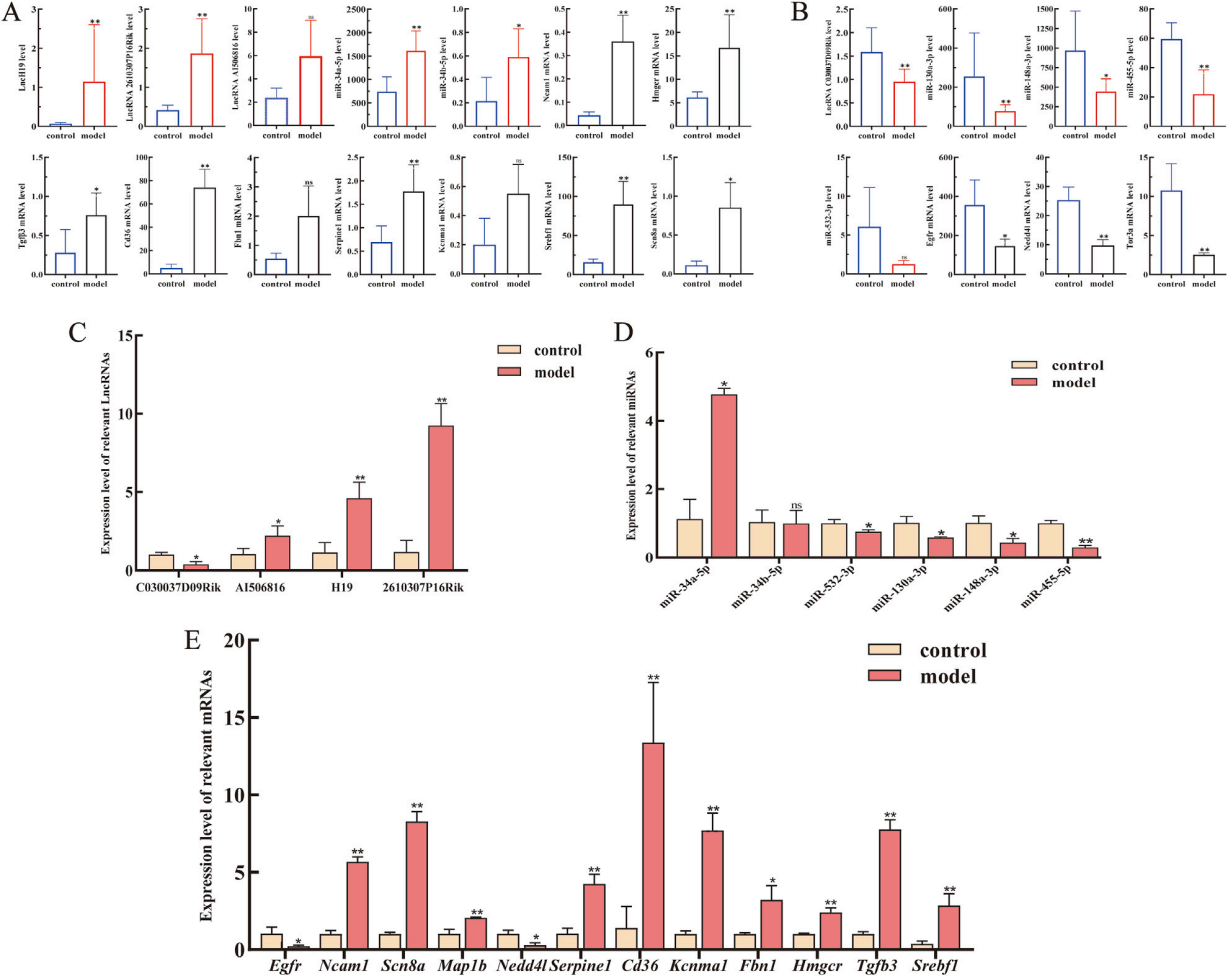
Expression levels of lncRNAs, miRNAs, and mRNAs from the core LMM network were validated by RT-qPCR. **(A)** Expression levels of upregulated lncRNAs, miRNAs, and mRNA from transcriptome sequencing data. **(B)** Expression levels of downregulated lncRNAs, miRNAs, and mRNA from transcriptome sequencing data. **(C–E)** Expression levels of lncRNAs, miRNAs, and mRNAs were verified by RT-qPCR. *P < 0.05 and **p < 0.01 vs. control; ns, not significant.

The expression landscape of the core ceRNA network, including four lncRNAs, six miRNAs, and 12 mRNAs of our interest (mRNAs selection ranked by degree), was chosen as the dataset for validation (Figures 5A, B). As the results described, compared to that in the control groups, lncRNA H19, lncRNA 2610307P16Rik, miRNA-34a-5p, miRNA-34b-5p, Hmgcr, Ncam1, and Scn8a were significantly upregulated in CCl_4_-treated C57BL/6 mice liver tissue, whereas lncRNA C030037D09Rik, miR-130a-3p, miR-148a-3p, miR-455-5p, miR-532-3p, Egfr, and Nedd4l were significantly downregulated. To verify the accuracy of sequencing data, RT-qPCR was subsequently used to validate the expression levels of these RNAs. As shown in Figures 5C–E, the expression of the 22 selected RNAs was generally consistent with the results of RNA-seq.

### 3.6 Core gene identification and validation in HSCs

**FIGURE 6:**
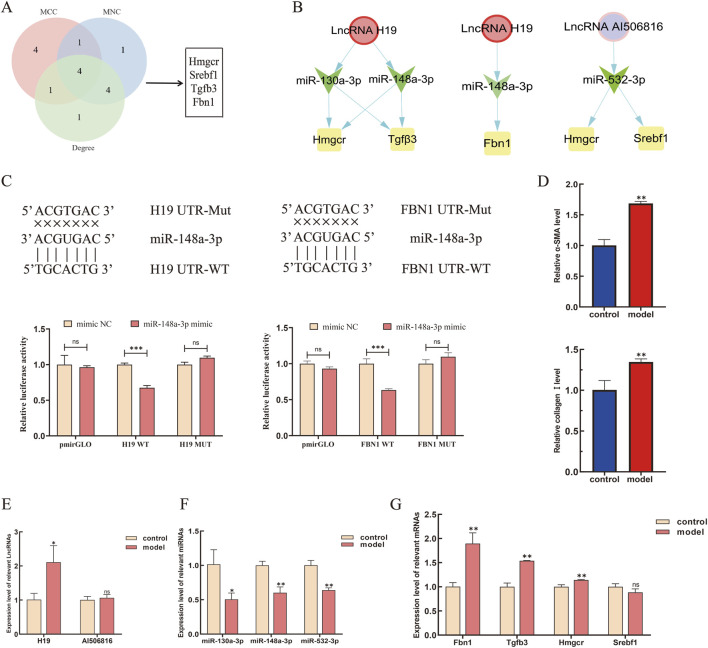
Expression levels of the core LMM network in HSCs were validated by RT-qPCR. **(A)** Screening of core genes by MCC, MNC, and degree algorithms. **(B)** Targeting relationship among the upregulated lncRNAs, miRNAs, and mRNAs were predicted, respectively. **(C)** lncRNA H19 and FBN1 were predicted as targets of miR-148a-3p by bioinformatic analysis. The luciferase activity of lncRNA H19 and FBN1 were declined in 293T-cells co-transfected with lncRNA-H19-3′UTR-WT, FBN1-3′UTR-WT, and miR-148a-3p mimic. **(D)** Expression level of α-SMA and collagen Ⅰ in HSCs. **(E–G)** Expression levels of core lncRNAs, miRNAs, and mRNAs were verified by RT-qPCR. *P < 0.05 and **p < 0.01. vs. control; ns, not significant.

Comprehensive topological evaluation of the PPI network’s 10 highest ranking nodes was conducted employing three centrality metrics (MCC, MNC, and degree). The results of the three algorithms were subsequently cross-analyzed, and four core genes were identified: 3-hydroxy-3-methylglutaryl coenzyme A reductase (HMGCR), sterol regulatory element-binding protein-1 (SREBP-1, SREBF1), transforming growth factor β3 (TGF-β3), and fibrillin-1 (FBN1) (Figure 6A). The expression of the four core genes is shown in Table 3, suggesting that the four core genes were all more upregulated in the model group compared to the control group. Finally, seven lncRNA–miRNA–mRNA pairs were obtained and shown in Figure 6B, namely two lncRNAs, three miRNAs, and four core mRNAs. Particularly, the results indicated that lncRNA H19 competitively binds miR-130a-3p and miR-148a-3p to regulate the involvement of Hmgcr, TGF-β3, and Fbn1 in the pathogenesis of LF. Similarly, lncRNA AI506816 regulates Hmgcr and Srebf1 by binding with miRNA-532-3p.

**TABLE 3:** Upregulated and downregulated expression of four mRNAs.

Gene ID	Log2(FC)	P-value	Regulation
HMGCR	1.23809660828731	0.0000420647850311634	Up
SREBF-1	2.32971781435332	1.18508292400436E-13	Up
TGF-β3	1.62991039192959	0.0197955454025826	Up
FBN1	1.77755333999216	5.15118223685786E-08	Up

Following bioinformatic prediction of the miR-148a-3p binding site, luciferase reporter assays were performed to validate the selective targeting of lncRNA H19 and FBN1 expression via competitive adsorption of miR-148a-3p. The luciferase activity results showed a significant decrease in activity for the wild-type FBN1 3′UTR (FBN1-wt) compared to that in the control group (p < 0.05), whereas no significant effect was observed for the mutant type (FBN1-mut). These findings suggest that miR-148a-3p can directly bind to specific sites within the FBN1 3′UTR and inhibit its expression (Figure 6C). Similarly, a notable reduction in luciferase activity was observed for H19-wt compared to its mutant counterpart (p < 0.05). To validate the ceRNA mechanism of the four core mRNAs in HSCs, TGF-β1 is used to induce HSC activation. In the current study, CCK-8 quantification of HSC proliferation 24 h post-treatment revealed a dose-dependent response to TGF-β1 stimulation (0.3125, 0.5, 1, 1.25, 2, and 2.5 ng/mL), with the dose-response profile demonstrating concentration-specific growth modulation (Supplementary Figure S2C). Then, RT-qPCR analysis demonstrated significant upregulation of α-SMA and collagen I expression in TGF-β1-activated JS-1 cells compared to that in unstimulated controls (Figure 6D). Finally, the gene expression of 7 ceRNA networks was validated in TGF-β1-induced JS-1 cells. As shown in Figures 6E–G, compared to the control groups, lncRNA H19, Fbn1, TGF-β3, and Hmgcr were significantly upregulated in TGF-β1-induced JS-1 cells and, as excepted, miR-130a-3p and miR-148a-3p were significantly downregulated, suggesting that five lncRNA–miRNA–mRNA relationship pairs, containing H19, miR-130a-3p, miR-148a-3p, TGF-β3, FBN1, and HMGCR, were involved in the activation of HSCs.

## 4 Discussion

LF is characterized as an abnormal wound-healing response elicited by persistent chronic liver injury (Aydın and Akçalı, 2018). The activation of resting HSCs, secreting extracellular matrix to destroy liver structure, is critical for the development of LF (Sun and Kisseleva, 2015). Therefore, the continuous elucidation of the HSC activation mechanism is of increasing significance for exploring the molecular processes of LF and screening for new diagnostic targets for LF. Recently, liver disease research has increasingly employed whole-transcriptome sequencing to delineate molecular pathology (Teng et al., 2024). In this research, employing whole-transcriptome RNA sequencing, we delineated dysregulated expression profiles of lncRNAs, miRNAs, and mRNAs in fibrotic liver tissues of CCl_4_-induced C57BL/6 mice.

Overall, 401 DE lncRNAs, 60 DE miRNAs, and 1,224 DE mRNAs, with significant differential expressed between control and model mice, were screened using whole transcriptome sequencing. Furthermore, bioinformatic analysis of the miRNA–lncRNA interaction network by the STARBASE miRNA interactome library was performed, thereby establishing a tripartite ceRNA regulatory axis containing DE miRNAs, lncRNAs, and mRNAs with functional convergence. Functional enrichment analyses (GO and KEGG) revealed that ceRNA-associated DE mRNAs exert their regulatory effects in LF pathogenesis primarily through pathway modulation and engagement in diverse metabolic processes. Finally, four core DE mRNAs were identified in the PPI network of lncRNA–miRNA–mRNA pairs, and a core lncRNA–miRNA–mRNA network was constructed consisting of four core mRNAs (HMGCR, SREBF-1, TGF-β3, and FBN1), two lncRNAs (lncRNA H19 and lncRNA AI506816), and three miRNAs (miR-130a-3p, miR-148a-3p, and miRNA-532-3p).

The expression of DE lncRNAs (lncRNA H19, lncRNA 2610307P16Rik, and lncRNA AI506816), DE miRNAs (miRNA-34a-5p and miRNA-34b-5p), and DE mRNAs (Hmgcr, Ncam1, and Scn8a) in the liver tissues of the C57BL/6 mice in the model group was significantly upregulated, whereas the expression of DE lncRNAs (lncRNA C030037D09Rik), DE miRNAs (miR-130a-3p, miR-148a-3p, and miR-455-5p, miR-532-3p), and DE mRNAs (Egfr and Nedd4l) was significantly downregulated. These results demonstrated that the RNA expression trends in RT-qPCR and RNA-seq were similar in C57BL/6 mice liver tissue. Subsequently, the expression of five core lncRNA–miRNA–mRNA pairs composed of four core genes (HMGCR, SREBF-1, TGF-β3, and FBN1) was confirmed in HSCs, which revealed that the expressions of lncRNA H19, miR-130a-3p, miR-148a-3p, miRNA-532-3p, HMGCR, TGF-β3, and FBN1 are closely related to HSC activation.

In this study, lncRNA C030037D09Rik and lncRNA AI506816 were identified as novel lncRNA, and they acted as a ceRNA, which were significantly correlated with LF progression. Among the four pivotal lncRNAs identified, 2610307P16Rik exhibited upregulated expression in C57BL/6 liver tissues treated with CCl_4_ *in vivo*. Notably, computational predictions suggested that lncRNA AI506816 could coordinate core regulatory genes (HMGCR and SREBF-1) with miR-532-3p via ceRNA mechanism. However, lncRNA AI506816 was not remarkably upregulated in experimental JS-1 cells when compared with that in controls, suggesting that lncRNA AI506816 may not correlate with HSC activation.

The lncRNA H19, an evolutionarily conserved RNA (murine chromosome 7/human 11p15.5) (Wakeling et al., 2017), has significant pathogenic associations with hepatic disorders, including metabolic-associated fatty liver disease (Errafii et al., 2021), cholestatic injury (Yu et al., 2023), and hepatocellular carcinoma (HCC) (Habashy et al., 2022). In particular, its specific involvement in the progression of LF has not been fully elucidated. H19 orchestrates multifaceted regulatory networks during LF through a bidirectional target regulation (Shi et al., 2024). In this study, we demonstrated that consistent H19 was increased in C57BL/6 liver tissues treated with CCl_4_ *in vivo* and in TGF-β1-activated HSCs *in vitro*. Furthermore, we used bioinformatic analysis and revealed H19’s ceRNA functionality, showing miRNA binding capacity (miR-148a-3p/130a-3p) and potential modulation of core regulatory targets HMGCR/TGF-β3/Fbn1. Moreover, the dual luciferase assay also validated the accuracy of our bioinformatic predictions. As reported by Zhu et al. (2019), H19-mediated modulation of TGF-β signaling via the miR-148a/USP4 axis has been identified, contributing to hepatic fibrogenesis progression. Thus, we hypothesized that lncRNA H19 functions as a molecular sponge for miR-148a-3p and miR-130a-3p to drive HSC activation through competitive binding of miRNA and downstream regulation of HMGCR, TGF-β3, and Fbn1 expression. However, further research on its ceRNA regulatory mechanisms in HSC activation is needed.

miRNAs are endogenous regulators of gene expression and play crucial roles in hepatic disease, and their specific expression may result in LF through the regulation of HSC activation (Zhou et al., 2024b). In this study, six hub miRNAs, including miRNA-34a-5p, miRNA-34b-5p, miR-130a-3p, miR-148a-3p, miR-455-5p, and miR-532-3p, were included in the ceRNA network and were also confirmed in fibrotic mouse liver tissue. Furthermore, out of the six hub miRNAs, the expression of miR-130a-3p, miR-148a-3p, and miR-532-3p was confirmed to be closely related to HSC activation. Previous research found that lncRNA Neat1 exerts ceRNA functionality in TGF-β1-activated HSCs by mediating Cyth3 regulation through miR-148a-3p sequestration (Huang et al., 2021). This regulatory pattern parallels our observed H19/miR-148a-3p/130a-3p axis modulating HMGCR/TGF-β3/FBN1 expression within the same cellular model. In addition, miR-130a-3p is also a key molecule involved in the HSC activation and apoptosis (Liu et al., 2021; Wang et al., 2017). Dysregulated miR-532-3p expression is implicated in HCC pathogenesis, whereas its functional role in LF remains under-characterized, and its expression in HSC activation is a novel finding of our study.

Extensive evidence confirms the pivotal role of TGF-β family members in orchestrating fibrotic remodeling. Injury-induced fibrotic microenvironment enhances TGF-β secretion and activates SMAD2/3 via TGF-β receptor I/II (TGFBR1/2) when an injury occurs (Budi et al., 2021). TGF-β3 is a ubiquitously expressed pleiotropic cytokine that regulates a wide range of organismal processes, including embryonic development, immunomodulation, cell cycle regulation, and fibrogenesis. TGF-β1 is a well-established key mediator of pro-fibrosis, whereas the regulatory role of TGF-β3 in the fibrotic process is complex and exhibits a dual effect. On the one hand, TGF-β3 can promote the development of LF and the deposition of an extracellular matrix; on the other hand, TGF-β3 has also been demonstrated to exert anti-fibrotic actions, potentially by modulating inflammatory responses or promoting matrix degradation (Liao et al., 2023; Jiang et al., 2021; Zhang et al., 2020). Specifically, the functions of TGF-β3 may evolve in response to the disease progresses. As reported by Wang et al. (2024), in the early stages of LF, the mRNA transcript levels and protein expression of TGF-β3 gradually increased, but when LF and pseudo-lobule were formed, it began to decline. Similarly, our study also observed increased expression of TGF-β3 in fibrotic tissues and TGF-β1-activated JS-1 cells. However, we only detected the increased expression levels of TGF-β3 in mouse liver tissues treated with CCl_4_ for 12 weeks, which may be associated with the progression of the fibrosis stage. Additionally, studies by Jinsheng Guo et al. (2021) and Tianhe Sun et al. (2021) also suggested that the expression of TGF-β3 in LF tissue was significantly higher than that in normal liver tissue. Conversely, Sun et al. (2024b) reported that targeted transient inhibition of TGF-β isoforms 2/3 ameliorated fibrosis *in vivo*, which suggests that TGF-β3 exerted anti-fibrotic actions. Therefore, these functional differences of TGF-β3 may stem from organ specificity in fibrosis and its dependence on the dynamic environmental changes occurring at different disease stages.

Fibrillin-1 (FBN1), one of the main constituents of microfibrils, is already known to have a role in LF. In this study, we observed FBN1 upregulation in both CCl4-induced fibrotic livers and TGF-β1-stimulated HSCs, which is aligned with the well-established mechanism that MFAP2 drives HSC activation through the FBN1/TGF-β/Smad3 axis (Sun et al., 2023). FBN1 was also shown to demonstrate functional coordination with ECM constituents, modulating TGF-β bioavailability to regulate connective tissue biomechanical properties (Summers et al., 2023). HMGCR has been reported to be involved in tumorigenesis in the early stage (Xiao et al., 2024). However, Zhang et al. (2025) suggested that the protein expression level of HMGCR is significantly elevated in fibrotic liver tissue and can be downregulated by antifibrotic drugs. In our study, HMGCR was upregulated in C57BL/6 liver tissues treated with CCl_4_ *in vivo* and in TGF-β1-treated HSCs *in vitro*, and this result is consistent with that of previous research.

These results indicated that lncRNA H19, miR-130a-3p, miR-148a-3p, miRNA-532-3p, HMGCR, TGF-β3, and FBN1 may participate in the activation of HSCs of LF and form a complex lncRNA–miRNA–mRNA network-regulatory relationship. However, our study had some limitations. First, the small sample size (n = 3) may limit the statistical power and increase the risk of false negative, potentially obscuring true effects. Furthermore, it may limit the generalizability of our findings. Future studies with larger cohorts are needed to validate our findings and provide more definitive mechanistic insights. Then, although the direct targeting relationships between miR-148a-3p and H19 and between miR-148a-3p and FBN1 were experimentally confirmed using dual-luciferase reporter assays, we were unable to extend this validation to other predicted ceRNA components within the network due to limitations in resources and scope. Future studies should employ additional luciferase assays and techniques such as RNA pull-down to comprehensively validate the entire ceRNA regulatory axis and further strengthen these findings. Finally, the regulatory effects on the protein levels of the target genes (TGF-β3, HMGCR, and FBN1) were not investigated. Due to space limitations, we cannot discuss the RNA molecular process in core ceRNA one by one. RT-qPCR showed that our analysis was reliable. We will further study the regulatory relationship of each key ceRNA and verify its relationship.

## 5 Conclusion

In conclusion, we identified aberrantly expressed lncRNAs, miRNAs, and mRNAs that were identified in CCl_4_-induced liver tissue with whole-transcriptome RNA sequencing and bioinformatic methods. An LMM regulatory network containing four lncRNAs, six miRNAs, and 148 mRNAs was constructed. We validated these lncRNAs, miRNAs, and 12 mRNAs with high score levels by RT-qPCR. Bioinformatic analysis illustrated promising ceRNA networks, along with biological processes and pathways involved in LF. In summary, this investigation systematically characterizes the lncRNA–ceRNA network, establishing a framework for understanding these transcripts’ potential pathogenic mechanisms in LF and facilitating biomarker discovery.

## Data Availability

The datasets presented in this study can be found in online repositories. The names of the repository/repositories and accession number(s) can be found in the article/Supplementary Material.

## Abbreviations

LF, liver fibrosis; HSCs, hepatic stellate cells; ECM, extracellular matrix; ceRNA, competing endogenous RNA; lncRNAs, long noncoding RNAs; circRNAs, circular RNAs; miRNAs, microRNAs; mRNAs, messenger RNAs; LMM, lncRNA–miRNA–mRNA; ncRNA, noncoding RNA; GO, Gene Ontology; KEGG, Kyoto Encyclopedia of Genes and Genomes; CCK-8, Cell Counting Kit-8; BP, biological processes; CC, cellular component; MF, molecular function.
