# Supplementary figures and images for: Construction of a HSC activation-related lncRNA–miRNA–mRNA ceRNA regulatory network reveals potential molecules involved in liver fibrosis
Source: Front Genet. 2025 Nov 10;16:1640326. doi: 10.3389/fgene.2025.1640326 (PMC12640689; doi:10.3389/fgene.2025.1640326)

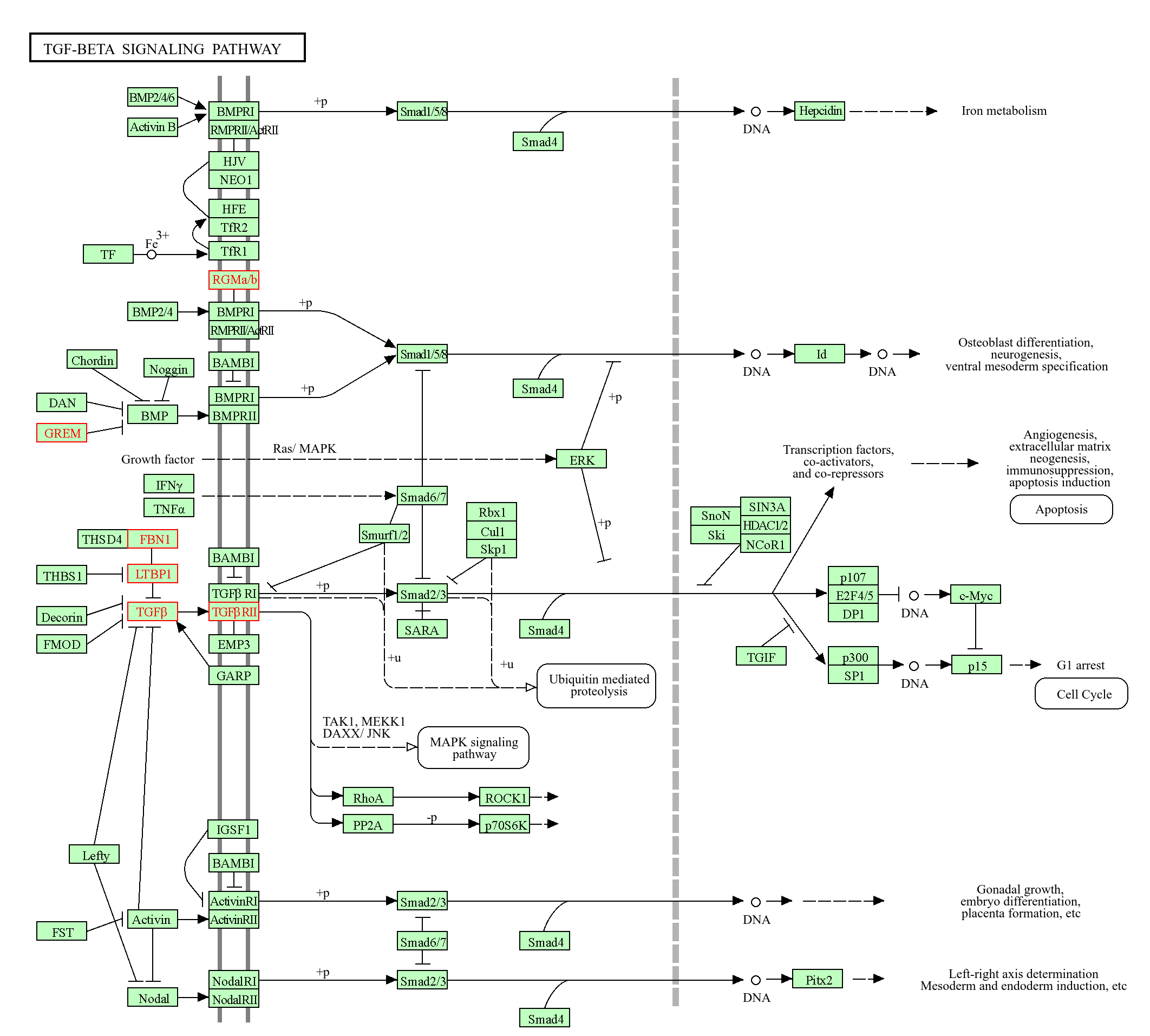

Supplement: Supplementary file 1 [file Image1.jpeg]

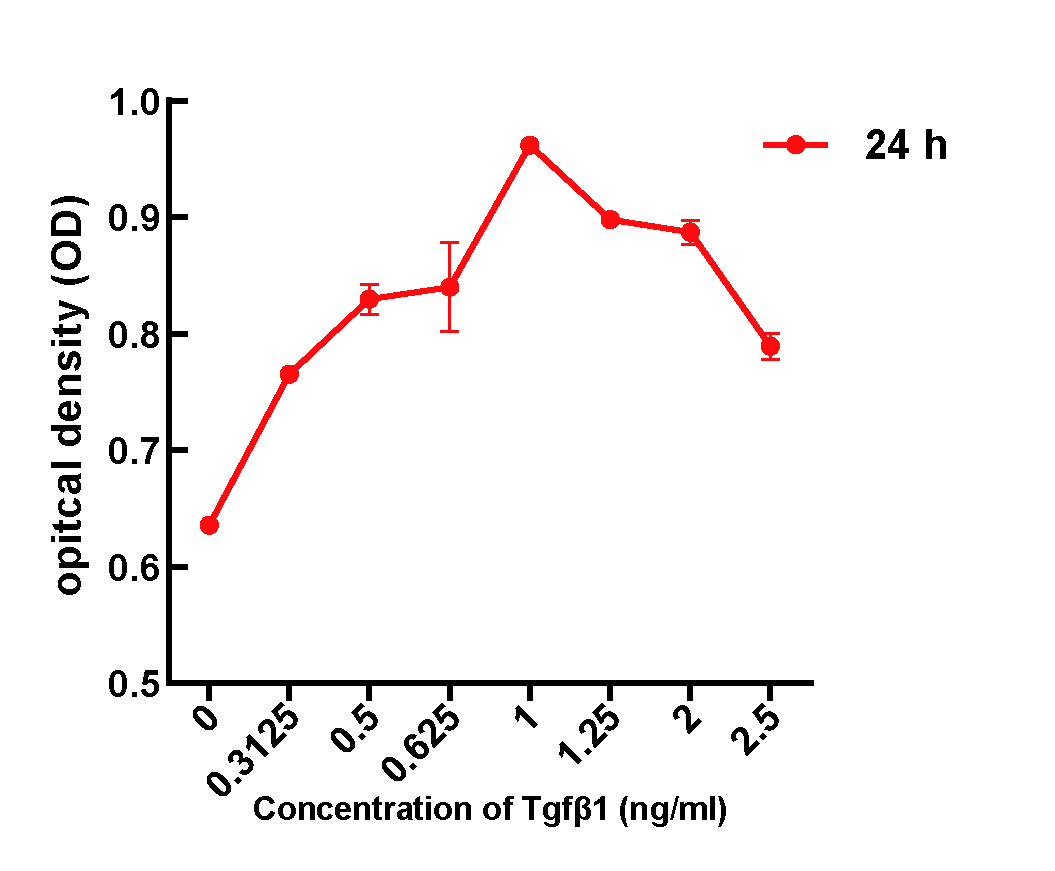

Supplement: Supplementary file 2 [file Image2.jpeg]
